# Wheelchair Servicing for Older Adults: Cross-Sectional Study

**DOI:** 10.2196/66472

**Published:** 2025-04-28

**Authors:** Anand Mhatre, Abigail Dumm, Muyun Zhao, Lorena Parra Rodriguez

**Affiliations:** 1 Division of Occupational Therapy, School of Health and Rehabilitation Sciences, College of Medicine, The Ohio State University, 406E, 453 W 10th Ave, Columbus, OH, 43210, United States, 1 6574640643; 2 Instituto Nacional de Geriatría, Mexico City, Mexico

**Keywords:** maintenance, mobile app, mobility, older adults, repair, seating, telemonitoring, wheelchair, wheelchair breakdown, wheelchair failure

## Abstract

**Background:**

Wheelchairs are assistive mobility devices known to experience frequent part failures and breakdowns within 6 months of regular use. No tools or technologies exist to monitor the wheelchairs’ condition or usage and inform stakeholders and users regarding when or how often they need to undergo servicing to avoid critical part failure.

**Objective:**

We aimed to test the association between wheelchair usage and manual wheelchair damage, part failures, and consequences for older wheelchair users and evaluate their preferences for a new wheelchair servicing technology.

**Methods:**

A cross-sectional study was performed with older manual wheelchair users atInstituto Nacional de Geriatría in Mexico. Demographic data, wheelchair information, failure counts, and preferences for new technology (sensor and smartphone app for servicing) were collected using surveys. Road shocks experienced by the wheelchair were collected for a week using a sensor.

**Results:**

Ten participants (mean [SD] age, 78.8 [11.8] y; 8 female and 2 male) participated. Seven experienced an average (SD) of 2.86 (1.36) self-reported part failures. Road shocks correlated with self-reported loose brake failures (*r(8)*=0.58, *P*=.09)*,* the damaged condition of tires (*r(8)*=0.61, *P=*.1), and the damaged condition of brakes (*r(8)*=0.58, *P*=.099)*.* No consequences were reported. Part failures increased as self-maintenance occurrences increased (*r(9)*=0.67, *P*=.04)*.* More than 8 participants responded that they would like to monitor the wheelchair’s condition using the new technology and purchase it at an average (SD) price of US $28.95 (18.13).

**Conclusions:**

In this study, the association between wheelchair usage and failures showed that data-driven wheelchair inspection schedules should be determined through a collaborative approach involving researchers and stakeholders in wheelchair repair provision and older adult users. Older adults are interested in using new technology to engage in wheelchair servicing.

## Introduction

Mobility is an essential element of an older person’s physical capacity. The loss of muscle mass and muscle strength, decreased flexibility, and problems with balance can all impair mobility [1]. Mobility impairment is found in 39% of people over 65 years of age, more than three times higher than that among the working population [2]. Wheelchairs enable mobility; however, these assistive mobility devices have a longstanding issue, that is, their parts break with little use.

Cross-sectional studies evaluating wheelchair durability in the community have reported manual wheelchair part failures within 2‐3 months of use [3]. They lead to physical, social, psychological, and economic consequences for the user [4] as well as device abandonment [5]. This problem is particularly severe in older adults; a higher incidence of failures was observed with elderly wheelchair users in a study conducted in El Salvador [6]. About 57% of the elderly experienced breakdowns in the past 3 months of wheelchair use due to high-risk failures of critical wheelchair parts such as casters and wheels. Around 75‐95% of the elderly participants rated their wheelchairs as unsatisfactory with unsafe working conditions, contributing to failure, breakdown, loss of mobility, and consequences.

In an effort to improve quality, the World Health Organization (WHO) Guidelines have recommended device quality testing using standards and routine follow-up with users, including the maintenance and repair of wheelchair parts [7]. However, this crucial area has been largely neglected. The maintenance checklists and programs developed by the WHO and research groups have enhanced users’ knowledge, performance, and compliance but have yet to prevent community failures [8]. Furthermore, a mixed-method study validating the user-led wheelchair servicing approach found that older adults using wheelchairs face numerous barriers to maintenance and repair, including health reasons, lack of tools, knowledge, and caregiver support [9]. This underscores the urgent need for novel strategies that can effectively prevent wheelchair failures and breakdowns in older adults.

For automobiles, advanced diagnostic technologies and odometer mileage direct maintenance and repair schemes. Similarly, low-cost sensors offer an opportunity. Using data loggers on community wheelchairs, studies have demonstrated a relationship between failure measures and wheelchair usage risk factors, such as wheelchair shocks and travel distance [10-12]. This study aims to validate this approach for older adult wheelchair users in Mexico. The study’s primary objective was to evaluate the relationship between wheelchair shocks and failure measures and the secondary objective was to evaluate older adult’s preferences for new servicing technology.

## Methods

### Study Location

The Ohio State University and Instituto Nacional de Geriatría (INGER) partnered to conduct a mixed-methods study with older adult wheelchair users in Mexico City.

### Ethical Considerations

The study was conducted in accordance with the Declaration of Helsinki and approved by the Research and Research Ethics Committees of theDirección de Investigación, Departamento de Investigación en Epidemiología Clínica at INGER, under the number DI-PI-009/2021 (date of approval August 11, 2021). Deidentified data transfer and use were permitted under a clinical study agreement between the two institutes. Written informed consent was obtained from all participants before beginning any test. The participants were reimbursed for their first visit travel to INGER.

### Study Participation and Recruitment

Participants who were full-time wheelchair users with manual wheelchair models and ≥60 years of age were screened and recruited. Sixty years of age is considered old age in international settings [13]. Flyers and word of mouth were approaches used to recruit older wheelchair users.

### Study Procedures

Between August and November 2021, participants underwent two study visits to the INGER facility in Mexico City to collect data. Survey administration, weight measurement, damage condition inspection using a validated Wheelchair Maintenance Assessment tool (W-MAT) [14], and sensor installation were conducted during the first visit. The W-MAT manual includes instructions for inspecting wheelchair parts and scores the damaged condition of parts on a Likert scale of 0‐2 with 0 indicating poor or damaged condition, 2 indicating good working condition, and NA indicating part not installed. Surveys included demographic information about the participants, part failures, maintenance, and consequences observed over the last 12 months. Participant’s preferences for smart wheelchair servicing technology, including interest in self-maintenance and receiving maintenance reminders and willingness to buy the technology, were gathered using Yes/No/Maybe responses through the survey.

During the study visit, the COVID pandemic was ongoing. The wheelchair-user population was considered vulnerable during the pandemic. Hence, disinfection and distancing guidelines were followed. The clinical visit room was cleaned prior to the visit. Only essential staff needed for data collection attended the participant and their accompanying caregiver, if present.

### Sensor Description

**Figure 1.**
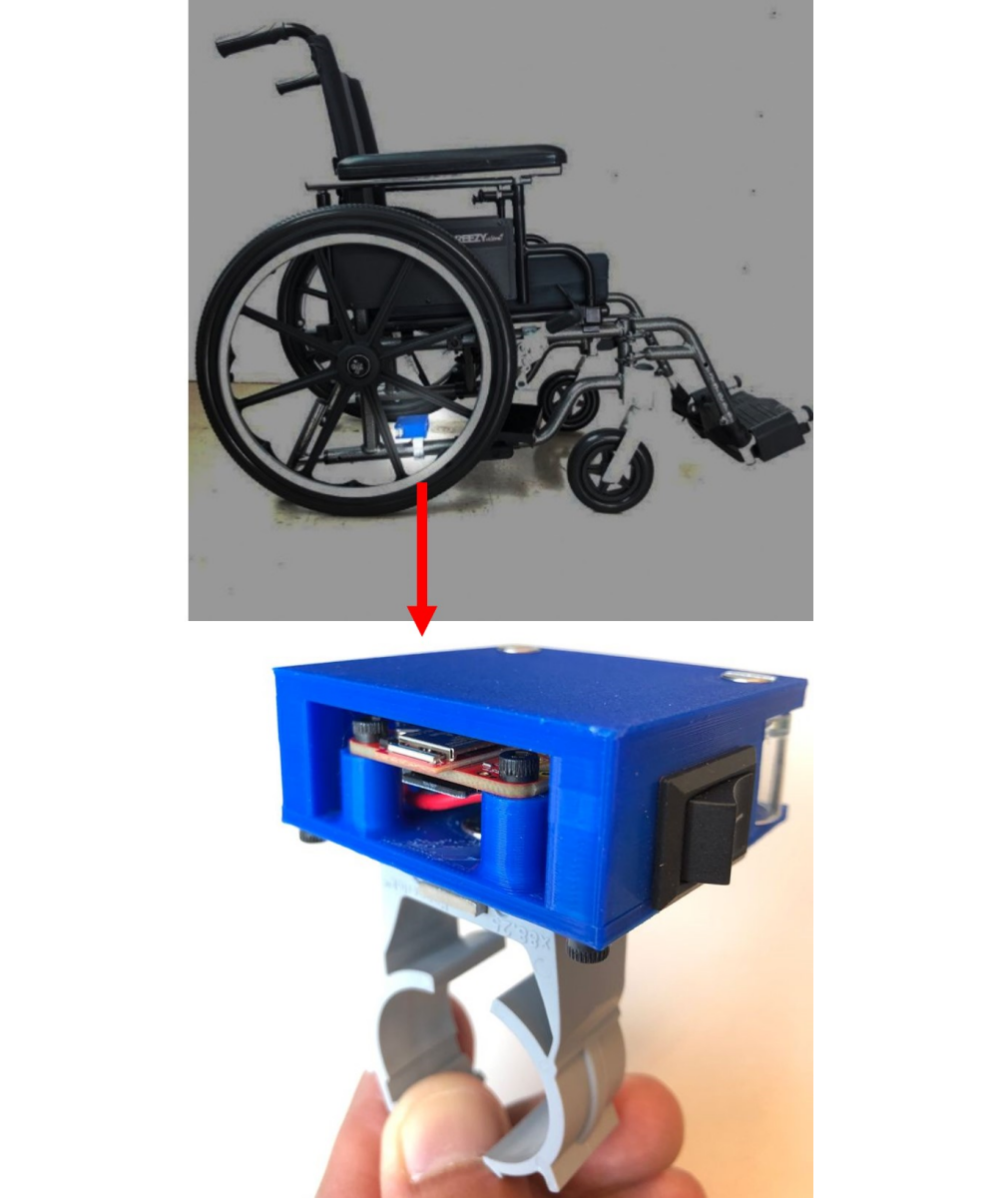
Wheelchair usage tracking sensor.

An inertial measurement unit sensor powered by three D-size alkaline batteries was installed on the side frame of the wheelchairs (Figure 1). The sensor is an upgrade to the sensor used in a previous study [15]; a tube clamp was attached for securing the sensor tightly on manual wheelchair tubular frames. The sensor stored the data on a micro-SD card. Participants collected sensor data for a week and returned for a second visit for sensor removal.

### Data Analysis

Descriptive statistics (mean [SD]) were performed on survey data. The normality of the data was tested using the Shapiro-Wilk test. For normal data, counts, means, and SD were computed. For non-normal data, medians and IQRs were computed. Shocks greater than or equal to five times the root mean square of the resultant acceleration value [15] were computed from sensor data. Maintenance patterns were analyzed. The frequency of maintenance reported by participants was ordered with no/not possible=0, daily=5, weekly=4, monthly=3, quarterly=2, and yearly=1, and a cumulative total maintenance score for each participant was computed. Pearson correlation analysis was used to compare shocks, failures, part condition scores, and maintenance patterns. Since this was a pilot testing study with a smaller sample size, a less conservative α-level of 0.1 was selected.

## Results

### Participant Characteristics

Ten attendant-propelled older adults using standard folding wheelchairs participated in the study. Their demographic characteristics are reported in Table 1. Seven participants experienced an average (SD) of 2.86 (1.36) self-reported part failures with tires, axles, wheels, casters, nuts or bolts, brakes, brakes, seat, and back supports. No consequences were reported.

**Table 1.** Demographic characteristics (N=10).

Characteristic	Value
Age, years, mean (SD)	78.8 (11.8)
Gender, n (%)	
Male	2 (20)
Female	8 (80)
Length of previous wheelchair usage, months, mean (SD)	36.8 (31.5)
Number of falls in the last year, median (IQR)	1 (0–2)^a^
Total weight (user and wheelchair), kg, median (IQR)	73.1 (66.3–105.05)
Weight on casters, kg, median (IQR)	23.2 (21.4–27.8)
Wheelchair weight, kg, median (IQR)	13.4 (13.2–16.4)
Maintenance score, mean (SD)	18.5 (17.25)^b^
Maintenance personnel, n (%)	
None	5 (50)
Caregiver	3 (30)
Technician	2 (20)
Days spent outside of the home environment, n (%)	
None	6 (60)
1–2	3 (30)
>5	1 (10)

a Only n=6 patients reported falls.

b The maintenance score was out of 125.

### Usage-Failure Relationships

Participants collected data for an average (SD) of 5.74 (4.34) days. One participant collected data for an hour and was omitted from the shock data analysis. Wheelchair shocks were on 40.38 (48.31) times/day with an average (SD) value of 7.89 (0.83) g. Shocks were correlated with self-reported loose brake failures (*r(8)*=0.58, *P*=.09) and tire damage condition *(r(8)*=0.61, *P*=.1)*.* Brake damage assessed using the W-MAT was associated with shocks (*r(8)*=0.58, *P*=.099)*.* Caster damage assessed using the W-MAT was moderately correlated with shocks but was not statistically significant (*r(8)*=0.52, *P*=.15)*.* The user’s age was correlated with seat failures (*r(7)*=0.58, *P*=.08)*.* Failures increased as maintenance occurrences increased (*r(9)*=0.67, *P*=.04).

### Servicing Technology Preferences

Six participants noted interest in self-maintenance, and 2 in collaboration with the caregiver. Seven participants said that they would like to receive reminders about wheelchair service. Nine participants said that they would like to monitor the wheelchair’s condition using the sensor and an app, and 8 participants said that they would like to buy the technology or use a mobile phone at an average (SD) price of US $28.95 (18.13).

## Discussion

### Principal Findings and Comparison With Previous Works

The findings from this study support the latest evidence on wheelchair usage-failure relationships and help drive the research efforts toward improving wheelchair reliability [11,12]. This study correlates road shocks with wheelchair part damage and failure measures experienced by older adults in less-resourced settings. Even though differences exist in wheelchair design, settings, materials, and demographic characteristics compared to previous studies with adults under 60 years old, usage dictates failure phenomena. The greater the usage, the more frequently servicing must happen. While the proportion of 70% experiencing at least one failure in 12 months is similar to the literature, a lower failure count among older adults is justified, an observation found with older power wheelchair users [10].

Users whose chairs received maintenance more regularly witnessed more failures, similar to previous studies with adults [11,12]. Hence, it is essential to consider a collaborative approach to wheelchair servicing where users can take up simple maintenance tasks such as those listed in the WHO maintenance training, and clinicians and technicians can perform complex inspections since professional-led inspections reduce failures [16,17]. Collaborative approaches such as mobile clinics and the engagement of community-based rehabilitation workers in follow-up are promoted in the WHO Prosthetics and Orthotics Standards and can be applied to wheelchairs [18]. As several groups and stakeholders look forward to reforming wheelchair servicing, this research emphasizes considering performance data in decision-making and approach development.

Older adults’ interest in using smart servicing technology coincides with a previous study in which 75% of participants upvoted new technology development, including smartphone apps [9]. This favorable outcome motivates the authors to continue developing new technologies while addressing barriers to wheelchair servicing reported by older adults. Following positive findings in this study, the authors aim to build a servicing intervention based on the usage-failure relationship and test its efficacy in preventing wheelchair failure.

### Limitations

The small sample size and use of a significance level of 0.1 for this pilot study increases the chance of a type 1 error. Hence, the authors aim to validate the usage-failure correlations in a larger cohort in future studies.

## Abbreviations

INGER: Instituto Nacional de Geriatría
WHO: World Health Organization
